# Neutralizing Activity of Broadly Neutralizing Anti-HIV-1 Antibodies against Clade B Clinical Isolates Produced in Peripheral Blood Mononuclear Cells

**DOI:** 10.1128/JVI.01883-17

**Published:** 2018-02-12

**Authors:** Yehuda Z. Cohen, Julio C. C. Lorenzi, Michael S. Seaman, Lilian Nogueira, Till Schoofs, Lisa Krassnig, Allison Butler, Katrina Millard, Tomas Fitzsimons, Xiaoju Daniell, Juan P. Dizon, Irina Shimeliovich, David C. Montefiori, Marina Caskey, Michel C. Nussenzweig

**Affiliations:** a Laboratory of Molecular Immunology, The Rockefeller University, New York, New York, USA; b Center for Virology and Vaccine Research, Beth Israel Deaconess Medical Center, Harvard Medical School, Boston, Massachusetts, USA; c Duke University Medical Center, Durham, North Carolina, USA; d Howard Hughes Medical Institute, Chevy Chase, Maryland, USA; Emory University

**Keywords:** broadly neutralizing antibodies, human immunodeficiency virus

## Abstract

Recently discovered broadly neutralizing antibodies (bNAbs) against HIV-1 demonstrate extensive breadth and potency against diverse HIV-1 strains and represent a promising approach for the treatment and prevention of HIV-1 infection. The breadth and potency of these antibodies have primarily been evaluated by using panels of HIV-1 Env-pseudotyped viruses produced in 293T cells expressing molecularly cloned Env proteins. Here we report on the ability of five bNAbs currently in clinical development to neutralize circulating primary HIV-1 isolates derived from peripheral blood mononuclear cells (PBMCs) and compare the results to those obtained with the pseudovirus panels used to characterize the bNAbs. The five bNAbs demonstrated significantly less breadth and potency against clinical isolates produced in PBMCs than against Env-pseudotyped viruses. The magnitude of this difference in neutralizing activity varied, depending on the antibody epitope. Glycan-targeting antibodies showed differences of only 3- to 4-fold, while antibody 10E8, which targets the membrane-proximal external region, showed a nearly 100-fold decrease in activity between published Env-pseudotyped virus panels and PBMC-derived primary isolates. Utilizing clonal PBMC-derived primary isolates and molecular clones, we determined that the observed discrepancy in bNAb performance is due to the increased sensitivity to neutralization exhibited by 293T-produced Env-pseudotyped viruses. We also found that while full-length molecularly cloned viruses produced in 293T cells exhibit greater sensitivity to neutralization than PBMC-derived viruses do, Env-pseudotyped viruses produced in 293T cells generally exhibit even greater sensitivity to neutralization. As the clinical development of bNAbs progresses, it will be critical to determine the relevance of each of these *in vitro* neutralization assays to *in vivo* antibody performance.

**IMPORTANCE** Novel therapeutic and preventive strategies are needed to contain the HIV-1 epidemic. Antibodies with exceptional neutralizing activity against HIV-1 may provide several advantages to traditional HIV drugs, including an improved side-effect profile, a reduced dosing frequency, and immune enhancement. The activity of these antibodies has been established *in vitro* by utilizing HIV-1 Env-pseudotyped viruses derived from circulating viruses but produced in 293T cells by pairing Env proteins with a backbone vector. We tested PBMC-produced circulating viruses against five anti-HIV-1 antibodies currently in clinical development. We found that the activity of these antibodies against PBMC isolates is significantly less than that against 293T Env-pseudotyped viruses. This decline varied among the antibodies tested, with some demonstrating moderate reductions in activity and others showing an almost 100-fold reduction. As the development of these antibodies progresses, it will be critical to determine how the results of different *in vitro* tests correspond to performance in the clinic.

## INTRODUCTION

Advances in B cell sorting techniques and microneutralization assays led to the discovery of broadly neutralizing antibodies (bNAbs) that have extensive breadth and potency against multiclade HIV-1 Env-pseudotyped virus panels (1–9). Preclinical experiments with mice and macaques demonstrated that the new antibodies can protect against and suppress active infection (10–17). Subsequent clinical trials have demonstrated the ability of bNAbs to suppress viremia (18–23) and delay viral rebound in humans (24, 25). On the basis of these experiments and early-phase clinical trials, bNAbs are being further investigated for use in the prevention and therapy of HIV-1. Their use in the clinic will depend in part on their breadth and potency against diverse HIV-1 strains, which have been evaluated primarily by using panels of HIV-1 Env-pseudotyped viruses representing the major genetic subtypes and circulating recombinant forms of the virus in diverse geographic locations. The vast majority of these molecularly cloned Env proteins are derived directly from plasma virus and were cloned by single-genome amplification to avoid recombination, although a minor subset was from peripheral blood mononuclear cell (PBMC)-derived virus (26–28). The use of Env-pseudotyped viruses produced in 293T cells, as opposed to primary isolates produced in PBMCs, allows the testing of large, diverse sets of clonal HIV-1 Env proteins with precisely known sequences in a controlled and highly reproducible manner (26, 29).

Despite the clear-cut advantages of using Env-pseudotyped viruses, experiments with anti-HIV-1 antibodies with limited breadth and potency revealed significant differences in sensitivity between the Env-pseudotyped viruses produced in 293T cells and parental uncloned viruses produced in PBMCs (26, 30). Molecular clones of viruses passaged in PBMCs were also shown to be less sensitive to neutralization by first-generation antibodies than the same viruses produced in 293T cells (26, 31). Whether these observations also apply to second-generation bNAbs that are currently being tested in the clinic has not yet been determined. Detailed formal comparisons of multiple assay formats for anti-HIV-1 antibodies have shown substantial differences in sensitivity and qualitative outcomes with a variety of serologic reagents. It has been recommended that multiple assays be used in parallel until a single assay emerges that best predicts clinical potency (32, 33).

Here we examine the ability of five current-generation bNAbs to neutralize circulating primary HIV-1 isolates and molecular clones produced in PBMCs and compare the results with those obtained with Env-pseudotyped viruses produced in 293T cells.

## RESULTS

From October 2014 through April 2017, 255 HIV-infected individuals had PBMCs isolated for viral outgrowth culture. Of the 255 outgrowth cultures performed, 184 were p24 positive after 4 weeks. One hundred fifty-five of these cultures were from patients on antiretroviral therapy (ART) who were virologically suppressed (viral load, <50 copies/ml) at the time of PBMC collection. The 29 viremic participants with p24-positive cultures were excluded from this analysis. At first, outgrowth culture isolates were only screened for sensitivity to 3BNC117. Over time, isolates were screened against additional antibodies. As a result, all 155 outgrown viruses were tested for 3BNC117 sensitivity. One hundred twenty-four isolates were screened for 10-1074 sensitivity, 95 were screened for VCR01 sensitivity, and 51 were screened for PGDM1400 and 10E8 sensitivity. The clinical characteristics of the 155 participants are shown in Table 1.

**TABLE 1:** Clinical characteristics of the 155 participants in this study

Characteristic	Value
No. (%) of:	
Males	138 (89)
Females	17 (11)
Age (yr)	
Median (IQR)^*a*^	45 (32–53)
Range	25–64
Race or ethnic group	
White non-Hispanic	29
Black non-Hispanic	51
Hispanic, regardless of race	43
Multiple	19
Other	7
Declined to answer	17
No. of CD4 T cells/mm^3^	
Median (IQR)	752 (605–911)
Range	258–1,743
Time (yr) since HIV diagnosis	
Median (IQR)	11 (5–18)
Range	1–34
Time (yr) on ART	
Median (IQR)	6 (3–15)
Range	1–27
No. (%) on ART	
Integrase inhibitor based	52 (34)
Protease inhibitor based	23 (15)
NNRTI^*b*^ based	70 (45)
Combination or other	10 (6)

a IQR, interquartile range.

b NNRTI, nonnucleoside reverse transcriptase inhibitor.

All of the participants included in this analysis were U.S. residents at the time of PBMC collection and were therefore likely to be infected with clade B viruses. Twenty-four participants tested by sequencing were indeed found to be infected with clade B viruses. Therefore, neutralization results from the PBMC-derived primary isolates were compared with those from the clade B pseudoviruses in the original panels used to characterize each antibody (2–5, 7).

**FIG 1:**
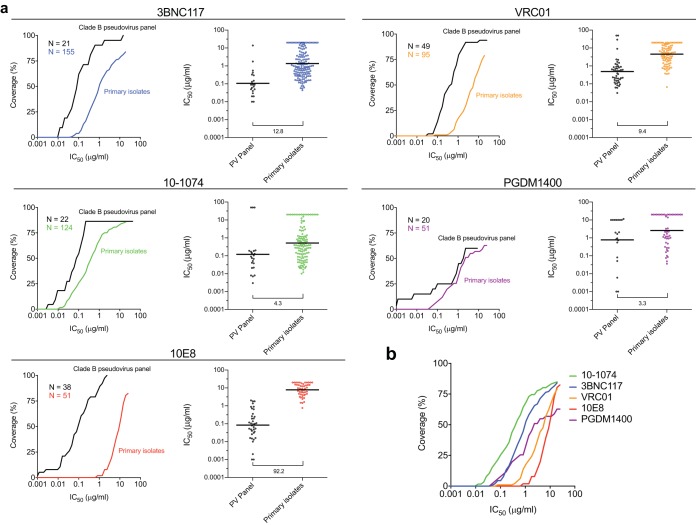
Breadth and potency of bNAbs against PBMC-derived primary isolates compared to those of original pseudovirus panels. (a) For each antibody, the graph on the left shows the percentage of viruses neutralized in the TZM-bl cell assay at a given IC_50_ (μg/ml) for the original clade B pseudovirus panels (black) and for PBMC-derived primary isolates (in color). N is the number of viruses tested in the original pseudovirus panel (black) and the PBMC-derived primary isolates (colored). The graph on the right shows the IC_50_ (μg/ml) for each isolate in the original pseudovirus (PV) panels and the PBMC-derived primary isolates as a dot. Black bars represent the geometric mean IC_50_s. The value under the bar below each dot plot is the fold difference in the geometric mean IC_50_ between the two groups. (b) Percentages of PBMC-derived primary isolates neutralized by the five bNAbs tested at a given IC_50_ (μg/ml) in the TZM-bl cell assay.

PBMC-derived primary isolates obtained from bulk outgrowth cultures were tested in the TZM-bl cell neutralization assay against five bNAbs that target different epitopes on the HIV-1 envelope: 3BNC117 and VRC01 (CD4 binding site), 10-1074 (V3 glycan), PGDM1400 (V1/V2 glycan), and 10E8 (membrane-proximal external region [MPER]). We found that every bNAb tested demonstrated less neutralization breadth and potency against the PBMC-derived primary isolates than against the original pseudovirus panels (Fig. 1a). Of note, the magnitude of this decline varied among the bNAbs tested, with the fold differences between the geometric mean 50% inhibitory concentrations (IC_50_s) of the two groups ranging from 3.3 for PGDM1400 to 92.2 for 10E8, which displayed the greatest disparity and was also the least potent against the primary isolates (Fig. 1b).

We next sought to account for the lesser bNAb breadth and potency observed against the PBMC-derived primary isolates than against the pseudovirus panels. Potential explanations included the following: (i) the PBMC-derived primary isolates were more resistant to neutralization than the viruses used to create the original pseudovirus panels; (ii) the PBMC-derived viruses, which were isolated from outgrowth cultures, consisted of a viral swarm, which increased resistance to neutralization; and (iii) production of viruses in 293T cells and/or the use of pseudoviruses results in increased sensitivity to neutralization.

**FIG 2:**
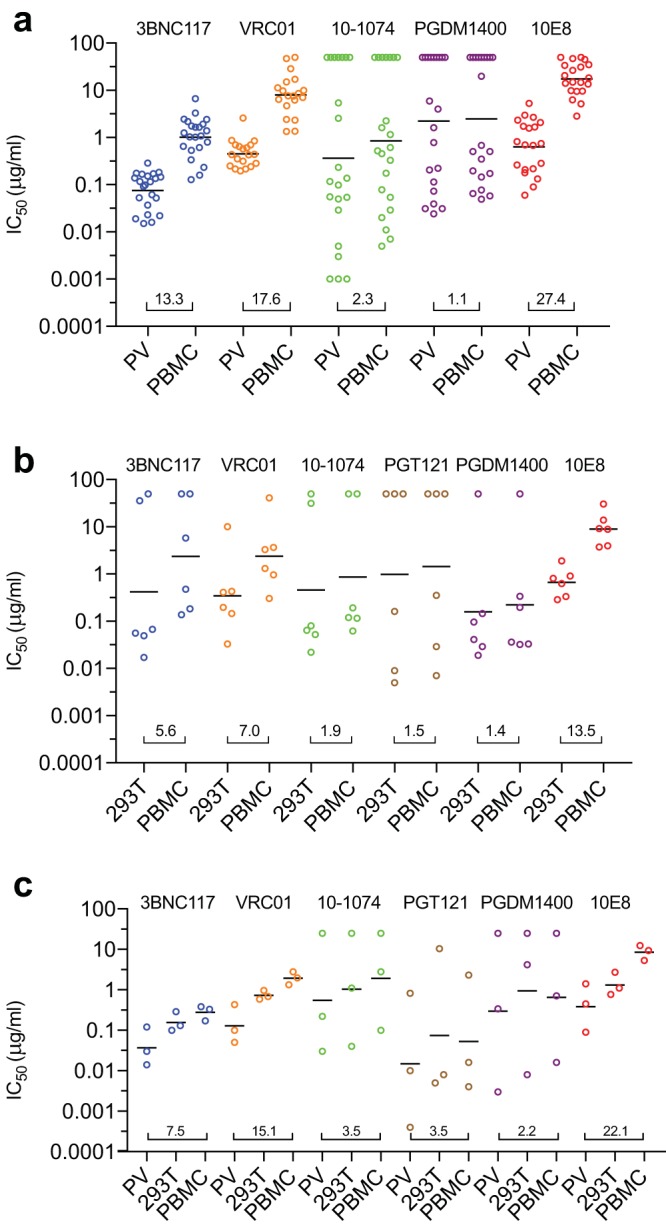
Neutralization sensitivity of clonal viruses produced as pseudoviruses, 293T-derived viruses, and PBMC-derived viruses. (a) The IC_50_s (μg/ml) of unique PBMC-derived outgrown clonal viruses (PBMC) and corresponding pseudoviruses (PV) are shown for each antibody. Each dot represents a single virus. Black bars represent the geometric mean IC_50_s. Values are the fold differences between the geometric mean IC_50_s for the two groups. (b) IC_50_s (μg/ml) for IMCs produced in 293T cells and PBMCs. Values are the fold differences between the geometric mean IC_50_s for the two groups. (c) IC_50_s (μg/ml) for IMCs produced as pseudoviruses (PV), in 293T cells, and PBMCs. Values are the fold differences between the IC_50_s for pseudoviruses and PBMC-derived viruses.

To determine whether the presence of viral swarms might account for the difference, we tested clonal PBMC-derived viruses and corresponding pseudoviruses. Clonal isolates were derived from four individuals chronically infected with clade B viruses. We used the quantitative and qualitative viral limiting-dilution outgrowth assay (Q^2^VOA) (34) to isolate unique PBMC-derived clonal viruses and produced corresponding pseudoviruses. The clonal nature of the viruses was confirmed by sequencing. Twenty-two unique PBMC-derived clonal viruses and corresponding pseudoviruses were produced and tested against the same five-member panel of bNAbs in the TZM-bl cell assay (Table 2). 10E8 again demonstrated the greatest difference between the geometric mean IC_50_s for the clonal PBMC-derived viruses and the corresponding pseudoviruses, with the PBMC-derived viruses demonstrating a 27.4-fold higher geometric mean IC_50_. The changes obtained with CD4 binding site antibodies 3BNC117 and VRC01 were 13.3- and 17.6-fold, respectively, while 10-1074 and PGDM1400, which target glycan-containing epitopes, demonstrated minimal fold changes (Fig. 2a). For viruses tested against 10-1074 and PGDM1400, there were rare instances in which IC_50_s for pseudoviruses were significantly higher than those for the corresponding PBMC-derived viruses (Table 2).

**TABLE 2:** IC_50_s in TZM-bl cells for PBMC-derived clonal isolates

Virus ID	IC_50_ (μg/ml)									
	3BNC117		VRC01		10-1074		PGDM1400		10E8	
	PV^*a*^	PBMC	PV	PBMC	PV	PBMC	PV	PBMC	PV	PBMC
106-1 2-9	0.053	0.650	0.696	14.996	0.005	0.020	5.956	0.173	2.661	45.083
106-1 1-2	0.037	0.336	0.282	4.716	0.050	0.454	0.205	0.348	1.678	49.577
106-1 2-8	0.061	0.535	0.428	7.547	0.054	0.328	0.031	0.077	1.718	20.645
106-1 E3	0.016	1.072	0.214	6.455	0.001	0.078	0.024	0.058	2.962	33.512
106-1 5-10	0.023	0.159	0.195	2.344	0.003	0.011	0.031	0.049	1.571	30.987
106-2 P12	0.015	0.128	0.214	1.350	<0.001	0.005	NT^*b*^	NT	5.308	34.973
106-2 P9	0.019	0.234	0.241	2.412	<0.001	0.007	0.039	0.065	2.323	47.310
199-1 AG1	0.146	3.295	0.352	28.761	0.117	1.158	0.781	0.677	0.820	9.475
199-1 AG7	0.052	0.611	0.439	11.249	0.055	0.524	0.073	0.196	0.698	26.392
199-1 AE5	0.288	2.169	0.316	47.027	0.098	0.615	1.613	0.146	0.681	14.000
199-1 AA4	0.022	1.017	0.250	8.536	0.137	2.264	0.223	0.503	2.072	50.000
199-2 BF12	0.184	6.611	0.864	>50	>50	0.054	>50	0.504	0.132	14.77
155-2 AK10	0.153	1.654	0.850	9.769	>50	>50	>50	>50	0.178	9.595
155-2 AD8	0.171	1.389	0.571	7.009	>50	>50	>50	>50	0.263	2.820
155-2 AS2	0.098	1.756	0.605	6.180	>50	>50	>50	>50	0.060	5.139
155-2 AS6	0.136	1.910	0.639	9.939	2.547	>50	>50	>50	0.218	6.265
155-2 AJ9	0.117	1.024	0.435	1.347	>50	0.029	>50	>50	0.090	13.562
155-2 AF6	0.174	1.649	0.693	7.617	>50	>50	>50	>50	0.285	9.823
155-1 F12	0.139	2.409	NT	NT	5.397	>50	>50	>50	NT	NT
155-1 K5	0.115	2.437	NT	NT	>50	>50	>50	>50	NT	NT
611 MI10	0.092	0.796	NT	NT	0.029	0.175	0.114	19.758	0.207	14.932
611 MH8	0.166	1.256	2.583	17.136	0.234	1.607	3.997	>50	0.749	18.267

a PV, pseudovirus.

b NT, not tested.

To determine whether the effects observed were due solely to the production of viruses in 293T cells versus PBMCs, we tested six infectious molecular clones (IMCs), five clade B and one clade C, that were first produced in 293T cells and then passaged once in PBMCs. These viruses were tested against the same five bNAbs, as well as PGT121 (Table 3). Once again, 10E8 demonstrated the greatest fold change in the geometric mean IC_50_ and the glycan-targeting antibodies demonstrated the smallest fold change (Fig. 2b). Compared to the testing performed with PBMC-derived primary clonal isolates and corresponding pseudoviruses, the fold differences between IMCs produced in 293T cells and those produced in PBMCs were generally smaller. PGT121, which targets the same epitope as 10-1074 (3), demonstrated a similar fold difference in its geometric mean IC_50_s.

**TABLE 3:** IC_50_s in TZM-bl cells for 293T- and PBMC-derived IMCs

bNAb	IC_50_ (μg/ml)											
	CH470		RHGA		CH058		CH077		CH164		MCST	
	293T	PBMC	293T	PBMC	293T	PBMC	293T	PBMC	293T	PBMC	293T	PBMC
3BNC117	35.614	>50	0.017	0.183	0.049	0.138	0.056	0.477	>50	>50	0.067	5.764
VRC01	10.005	40.859	0.033	0.304	0.197	0.958	0.146	1.313	0.429	3.667	0.411	3.298
PGDM1400	>50	>50	0.162	0.353	>50	>50	0.005	0.007	0.009	0.029	>50	>50
10-1074	0.080	0.192	>50	>50	0.022	0.062	31.389	>50	0.064	0.120	0.052	0.115
PGT121	0.029	0.032	0.146	0.195	0.019	0.036	>50	>50	0.096	0.064	0.041	0.033
10E8	0.288	3.722	0.804	8.904	0.909	3.974	0.628	13.959	1.896	30.348	0.333	9.133

To directly compare pseudoviruses to corresponding full-length molecular clones, we produced three IMCs, two clade B and one clade C, as 293T-derived pseudoviruses, 293T-derived IMC viruses, and PBMC-derived IMC viruses and tested them against the same six bNAbs. Overall, pseudoviruses demonstrated greater sensitivity to neutralization than IMC viruses produced in 293T cells, while IMC viruses produced in PBMCs were the most resistant to neutralization (Fig. 2c). While pseudoviruses were generally the most sensitive to neutralization and PBMC-derived viruses were generally the most resistant to neutralization, this was not the case for every antibody-virus pair tested (Table 4). We conclude that pseudoviruses produced in 293T cells generally exhibit even greater increases in sensitivity to neutralization than IMCs produced in the same cells.

**TABLE 4:** IC_50_s in TZM-bl cells for IMCs produced as pseudoviruses, in 293T cells, and in PBMCs

bNAb	IC_50_ (μg/ml)								
	Ce1086			RHPA			WITO		
	PV^*a*^	293T	PBMC	PV	293T	PBMC	PV	293T	PBMC
3BNC117	0.12	0.29	0.93	0.014	0.1	0.17	0.03	0.13	0.38
VRC01	0.43	0.97	2.8	0.05	0.68	1.33	0.1	0.59	1.98
10-1074	>25	>25	>25	0.03	0.04	0.1	0.22	1.1	2.8
PGT121	<0.0004	0.005	0.004	0.01	0.008	0.016	0.82	10.4	2.3
PGDM1400	>25	>25	>25	0.34	4.2	0.71	0.003	0.008	0.016
10E8	0.45	2.7	12.4	1.4	0.77	9.3	0.09	1.1	5.3

a PV, pseudovirus.

In addition to the virus-producing cell, the target cell utilized in a neutralization assay can affect neutralization sensitivity, with PBMC-based assays generally reporting decreased neutralization sensitivity and higher variability than cell line-based assays (32, 35). However, there are also reports of increased neutralization sensitivity in PBMC assays (36). To determine if the changes in neutralization sensitivity between viruses produced in 293T cells and PBMCs were related to the use of the TZM-bl cell line as the target in the neutralization assay, three IMCs produced in 293T cells and then passaged once in PBMCs were tested in a PBMC-based assay. We found that, like the TZM-bl cell assay, the PBMC assay also reported greater neutralization sensitivity for viruses produced in 293T cells (Tables 5 and 6).

**TABLE 5:** IC_50_s in PBMCs for IMCs tested in a PBMC neutralization assay

bNAb	IC_50_ (μg/ml)					
	Ce1086		RHPA		WITO	
	293T cells	PBMCs	293T cells	PBMCs	293T cells	PBMCs
3BNC117	0.09	1.4	0.06	0.04	0.14	0.23
VRC01	1.6	8.0	0.43	1.7	0.9	3.7
10-1074	>25	>25	0.007	0.017	0.68	5.6
PGT121	0.02	0.016	0.005	0.015	6.1	23.4
PGDM1400	>25	>25	0.2	0.16	0.003	0.016
10E8	2.3	8.1	1.22	6.3	0.61	7.8

**TABLE 6:** Fold differences in geometric mean IC_50_s for IMCs produced in PBMCs and 293T cells and tested in TZM-bl cell and PBMC assays

bNAb	Fold difference between geometric mean IC_50_s (IMC-PBMC/IMC-293T) for indicated assay type	
	TZM-bl cells	PBMCs
3BNC117	1.8	2.6
VRC01	2.7	4.3
10-1074	1.9	2.7
PGT121	0.7	2.1
PGDM1400	0.8	1.6
10E8	6.4	6.1

To determine if the fold differences between IC_50_s are similar for bNAbs that target the same epitope, the three IMCs tested as pseudoviruses and as PBMC-derived viruses were also tested against additional bNAbs targeting the CD4 binding site, V1/V2 glycan, V3 glycan, or the MPER (Table 7). Of the antibodies tested, the MPER-targeting antibodies demonstrated the greatest IC_50_ fold differences, while the V1/V2 glycan-targeting antibodies had the smallest fold differences. The difference between the geometric mean IC_50_s of the CD4 binding site-targeting antibodies and the V1/V2 glycan-targeting antibodies and the difference between the MPER-targeting antibodies and the V1/V2 glycan-targeting antibodies were statistically significant (*P* = 0.001 and *P* = 0.0006, respectively [Kruskal-Wallis test and Dunn multiple-comparison test]). These results suggest that the increase in neutralization sensitivity observed for 293T-derived pseudoviruses compared to PBMC-derived viruses similarly affects antibodies that target the same epitope.

**TABLE 7:** Fold differences in IC_50_s for IMCs produced in PBMCs or as pseudoviruses by epitope

Epitope and bNAb	Fold difference in IC_50_ (IMC-PBMC/pseudovirus)			Mean fold change per:	
	WITO	RHPA	Ce1086	Antibody	Epitope
CD4bs					
3BNC117	12.7	12.1	7.8	10.6	12.1
VRC01	19.8	26.6	6.5	15.1	
VRC07-523	10.3	15.9	8.3	11.1	
V1/V2 glycan					
PG9	1.0	<0.3	R^*a*^	<1.0	<0.8
PG16	0.3	0.1	R	0.2	
PGDM1400	5.3	2.1	R	3.3	
V3 glycan					
10-1074	12.7	3.3	R	6.5	4.1
PGT121	2.8	1.6	10.0	3.6	
PGT128	R	2.4	R	2.4	
MPER					
10E8	58.8	6.6	27.6	22.0	>23.3
4E10	>15.6	R	>25	>19.7	
2F5	>38.5	R	R	>38.5	

a R, pseudovirus resistant to bNAb (IC_50_, >20 μg/ml).

For most of the antibodies tested, the decline in neutralization sensitivity we observed against PBMC-derived primary isolates compared to the original pseudovirus panels appears to be a result of the difference in neutralization sensitivity between pseudoviruses and PBMC-derived viruses (Table 8). For 10E8, the difference in neutralization sensitivity between pseudoviruses and PBMC-derived viruses only partially explains the large difference observed between the original pseudovirus panel and the PBMC-derived primary isolates. For this antibody, it is possible that the original pseudovirus panel included highly sensitive clade B strains not representative of currently circulating clade B viruses, resulting in an even greater overestimation of the antibody's breadth and potency against PBMC-derived primary isolates.

**TABLE 8:** Decreased neutralization sensitivity of PBMC-derived isolates compared to pseudoviruses

bNAb	Fold difference between geometric mean IC_50_s		
	Primary isolates vs original pseudovirus panels	Clonal isolates vs corresponding pseudoviruses	PBMC-derived IMCs vs corresponding pseudoviruses
3BNC117	12.8	13.3	7.5
VRC01	9.4	17.6	15.1
10-1074	4.3	2.3	3.5
PGDM1400	3.3	1.1	2.2
10E8	92.2	27.4	22.1

## DISCUSSION

Env-pseudotyped virus panels for HIV-1 neutralization assays were designed to standardize the measurement of neutralizing activity of serum and monoclonal antibodies. Initial testing revealed that pseudoviruses produced in 293T cells were considerably more sensitive to neutralization by patient serum and first-generation anti-HIV-1 monoclonal antibodies than were viruses produced in PBMCs (26). The results presented here are consistent with earlier reports showing that the TZM-bl cell assay with Env-pseudotyped virus is among the most sensitive and reproducible of multiple assays evaluated for HIV-1 neutralization (32, 33). While this superior sensitivity and extended range of detection are desirable features, assays of 293T-grown Env-pseudotyped viruses in TZM-bl cells may also have the potential to overestimate clinical potency. Correlations between the *in vitro* activity and clinical potency of HIV-1 bNAbs have only been possible to date when using experimental challenges of nonhuman primates and transgenic mice. Results indicate that *in vivo* potency against the acquisition of infection in these passive-transfer models is related to antibody potency and the challenge dose used (11, 17). A clearer picture may emerge from ongoing therapy and prevention trials with bNAbs in humans (ClinicalTrials.gov registration no. NCT02825797, NCT02588586, NCT02568215 [HVTN 703/HPTN 081], and NCT02716675 [HVTN 704/HPTN 085] www.ampstudy.org) (18, 19, 25). Consistent with the recommendations of the NeutNet reports (32, 33), we recommend that multiple assays be employed when assessing neutralization as a correlate of infection risk and when defining protective titers in these human clinical trials. Through these efforts, a more accurate picture may emerge to help identify the best assay and the most relevant levels of potency of each antibody in each assay that correlate with clinical outcomes.

While the relevance of *in vitro* neutralization testing to *in vivo* antibody performance in clinical trials remains to be defined, clinical trials conducted to date evaluating VRC01 and 3BNC117 in the setting of treatment interruption suggest that PBMC-derived viruses may be more predictive than pseudoviruses. In the VRC01 A5340 study, baseline participant pseudoviruses showed a geometric mean IC_80_ of 3.52 μg/ml (24). Assuming a 1-log difference between pseudoviruses and PBMC-derived viruses for VRC01, this would mean a geometric mean IC_80_ of 35.2 μg/ml. The median time to rebound with three infusions of VRC01 was 4 weeks, only a small delay compared to historical controls, suggesting that most of the participants were, in fact, resistant. In contrast, participants in the 3BNC117 study had baseline viruses with a geometric mean IC_80_ of 1.12 μg/ml; however, this IC_80_ was for PBMC-derived isolates, not pseudoviruses. Unlike the study evaluating VRC01, participants administered 3BNC117 experienced an average delay in rebound of 6.7 weeks after two infusions and 9.9 weeks after four infusions (*P* < 0.00001 versus historical controls) (25). Further studies are required to definitively ascertain whether PBMC-derived viruses are better predictors of *in vivo* activity than pseudoviruses in the context of both therapy and prevention. This would be especially important for antibodies like 10E8 that show the highest level of disparity between PBMC-derived viruses and pseudoviruses.

Our data indicate that circulating PBMC-derived primary isolates are significantly less sensitive to current-generation anti-HIV-1 monoclonal antibodies than the pseudoviruses utilized as standard reference reagents in the TZM-bl cell assay. This discrepancy is largely due to the greater sensitivity of viruses produced in 293T cells than viruses produced in PBMCs, with an additional contribution of the pseudotyped virus backbone versus the full-length virus. Moreover, the difference in neutralization sensitivity between PBMC-derived viruses and pseudoviruses differs among antibodies, with 10E8 exhibiting the greatest difference and the glycan-targeting antibodies being affected the least. Similarly, 4E10, a first-generation MPER-targeting bNAb, was found to exhibit the greatest discrepancy in neutralization among first-generation bNAbs when tested against pseudoviruses and PBMC-derived viruses (26).

There are a number of potential explanations for why 293T-derived pseudoviruses exhibit greater sensitivity to neutralization. First, 293T cells are not the natural host of HIV-1 and viruses produced in these cells may differ from viruses produced in T cells in either the pattern or the heterogeneity of glycoforms (37–39). Second, neutralization sensitivity may also depend on the number of Env spikes on the virion, which may differ between viruses produced in 293T cells and viruses produced in PBMCs (31). Third, our observation that pseudoviruses are somewhat more sensitive to neutralization than IMCs produced in the same 293T cells suggests that the pseudovirus backbone can also influence sensitivity to antibody neutralization *in vitro* (40).

In summary, we found that current bNAbs demonstrate significantly less breadth and potency against circulating PBMC-derived primary isolates than against the original pseudovirus panels. This effect appears to be due to the fact that pseudoviruses are more sensitive to neutralization by current generation bNAbs than PBMC-derived viruses. Furthermore, the magnitude of this effect can vary, depending on the antibody. A majority of the viruses we tested were clade B. Therefore, these findings need to be confirmed with isolates from additional clades. As the clinical development of anti-HIV-1 bNAbs progresses, it will be critical to determine the relevance of *in vitro* neutralization assays to *in vivo* antibody performance.

## MATERIALS AND METHODS

### Study participants.

All participants were recruited at The Rockefeller University Hospital, New York, NY, through protocol MCA-823, which was approved by The Rockefeller University Institutional Review Board. All participants provided written informed consent. Participants were HIV-infected adults 18 to 65 years old in general good health and with laboratory results that would qualify them for one of The Rockefeller University's clinical trials evaluating bNAbs 3BNC117 and/or 10-1074. Only participants residing in the United States and virologically suppressed on ART were included in this analysis.

### PBMC bulk outgrowth culture.

PBMC bulk outgrowth culture was performed as previously described (18, 19). Briefly, healthy donor PBMCs were obtained by leukapheresis under study protocol MNU-0628 at The Rockefeller University. All donors provided written informed consent before participation. Healthy donor PBMCs were stimulated at a density of 5 × 10^6^/ml in Iscove's modified Dulbecco's medium or RPMI containing 10% fetal bovine serum, 1% penicillin-streptomycin, and phytohemagglutinin (PHA) at 1 μg/ml for 2 or 3 days at 37°C and 5% CO_2_. PBMCs were obtained from HIV-infected individuals, and CD4^+^ T lymphocytes were isolated by negative selection with magnetic beads (Miltenyi). A total of 5 × 10^6^ of the stimulated CD8-depleted healthy donor PBMCs were then cocultured with 5 × 10^6^ to 10 × 10^6^ patient CD4^+^ T cells at 37°C and 5% CO_2_. Irradiated heterologous PBMCs (1 × 10^7^) were also added. The medium was replaced weekly, and the presence of p24 in the culture supernatant was quantified by the Alliance HIV-1 p24 antigen enzyme-linked immunosorbent assay kit (PerkinElmer) or the Lenti-X p24 Rapid Titer kit (Clontech). The infectivity of virus cultures was confirmed by a 50% tissue culture infective dose assay with TZM-bl cells (26).

### Clonal viruses.

HIV-1 IMCs were kindly provided by Beatrice Hahn. 293T cells were transfected to generate 293T-derived infectious virus, which was then passaged once through human PBMCs. Additional clonal viruses were isolated from four individuals infected with clade B HIV-1 by using Q^2^VOA. Q^2^VOA is an outgrowth assay that isolates replication-competent viruses from the latent reservoir by a limiting-dilution method such that each virus likely originates from a single reactivated infectious provirus (34). Q^2^VOA was performed as previously described (34) to obtain unique clonal PBMC-derived viruses. Full-length *env* was sequenced, and pseudoviruses were produced in 293T cells.

### Neutralization assays.

Viruses were tested against bNAbs by using the TZM-bl cell neutralization assay as previously described (26, 41). A subset of assays was also performed with PHA-stimulated target PBMCs pooled from six individual donors. PBMC assays utilized Env.IMC.LucR viruses and a reduction in Renilla luciferase reporter gene expression to measure neutralization as previously described (35). Neutralization assays were conducted in laboratories meeting Good Clinical Laboratory Practice quality assurance criteria. For some bulk outgrowth culture primary isolates tested against 3BNC117, VRC01, and 10-1074, the maximum antibody concentration tested was 20 μg/ml, as opposed to the maximum PGDM1400 and 10E8 concentration of 50 μg/ml tested against all isolates. Therefore, a value of >20 μg/ml is used in the analysis instead of the reported titer of >50 μg/ml for all of the bulk outgrown primary isolates tested.

### Pseudovirus production in 293T cells for clonal outgrowth isolates.

Pseudovirus production was performed as previously described (42). Briefly, the cytomegalovirus (CMV) promoter was amplified by PCR from the pcDNA 3.1D/V5-His-TOPO plasmid (Life Technologies) with forward primer 5′-AGTAATCAATTACGGGGTCATTAGTTCAT-3′ and reverse primer 5′-CATAGGAGATGCCTAAGCCGGTGGAGCTCTGCTTATATAGACCTC-3′. A 1-μl volume of the first-round PCR product from each individual *env* gene obtained from Q^2^VOA cultures was amplified with primers 5′-CACCGGCTTAGGCATCTCCTATGGCAGGAAGAA-3′ and 5′-ACTTTTTGACCACTTGCCACCCAT-3′. PCR products were purified with the Macherey-Nagel Gel and PCR purification kit. The CMV promoter amplicon was fused to individual *env* genes by overlap PCR with 10 ng of envelope and 0.5 ng of CMV with forward primer 5′-AGTAATCAATTACGGGGTCATTAGTTCAT-3′ and reverse primer 5′-ACTTTTTGACCACTTGCCACCCAT-3′. Resulting amplicons were analyzed by gel electrophoresis, purified with the Macherey-Nagel Gel and PCR purification kit, and cotransfected with pSG3Δ*env* into HEK293T cells to produce pseudoviruses as previously described (42).
